# Tailoring Safety Training Material to Migrant Farmworkers: An Ergonomic User-Centred Approach

**DOI:** 10.3390/ijerph17062104

**Published:** 2020-03-22

**Authors:** Federica Caffaro, Giorgia Bagagiolo, Margherita Micheletti Cremasco, Lucia Vigoroso, Eugenio Cavallo

**Affiliations:** 1 Department of Education, Roma Tre University, 00185 Rome, Italy; 2 Institute for Agricultural and Earthmoving Machines (IMAMOTER), National Research Council of Italy (CNR), 10135 Torino, Italy; g.bagagiolo@ima.to.cnr.it (G.B.); l.vigoroso@ima.to.cnr.it (L.V.);; 3 Department of Life Sciences and Systems Biology, University of Torino, 10123 Torino, Italy; margherita.micheletti@unito.it

**Keywords:** agriculture, ergonomics, migrant farmworker, occupational safety, training intervention

## Abstract

Lectures are the most commonly used training method but may not be effective in communicating safety information among migrant workers because of language and cultural barriers. The comprehension of safety information is particularly urgent in highly hazardous sectors such as agriculture, which has a high migrant workforce rate. In this study, an ergonomic user-centred approach was adopted to develop and evaluate safety training material targeting migrants employed on Italian farms. Focus groups with both trainers and migrants were carried out to collect information on critical issues in traditional training material, the most and least comprehended topics and suggestions for training improvement. Based on the focus group analysis, the training material was developed considering several visual factors and more engaging interaction between trainers and migrant trainees. The usability of the developed material was then evaluated in a group of 20 migrant farmworkers through a questionnaire assessing the knowledge of safety information before (T0) and after (T1) the training (effectiveness), perceived effort in learning (efficiency) and user satisfaction after training. The results showed improved knowledge, low perceived effort and high satisfaction, pointing to the positive effects of active user involvement when designing targeted training materials.

## 1. Introduction

Training has been acknowledged as a key factor in the promotion of occupational safety and health (OSH) in different productive sectors [1,2,3]. Training makes workers aware of the hazards they may encounter in the workplace, informs them of the tools and regulations that are in place to protect them and pushes them towards safe behaviours [4]. A number of studies have found that many safety training methods are used (i.e., classroom activities, hands-on demonstrations or 3D simulations) [5,6,7], which could be more or less passive (lectures and computer-based programs) or particularly engaging (hands-on demonstrations) [1].

Despite its low level of engagement, the most commonly used safety training method is based on lectures [8], in which the trainer explains to workers the concepts of risk, hazard and injury and how to prevent them in the workplace. To increase training engagement, trainers may encourage feedback from trainees, testing their comprehension of the provided information and involving them in checking the accuracy of the answers (i.e., a moderately engaging method training [1]). Visual tools are often used to increase trainees’ comprehension of the safety information explained since such tools are known to have a stronger emotional impact and to communicate more information in a limited space compared to written text [6].

When safety training is administered to workers from different countries, the difficulty in the comprehension of the safety material used by trainers during lectures should be carefully considered. Indeed, the diversity of the languages, cultures and education of migrant workers often prevent them from understanding OSH rules and correct practices [9], which may lead to the adoption of incorrect and unsafe behaviours, increasing the risk of incidents and injuries [10]. During intercultural OSH lectures, the trainer is often supported by visual tools such as maps, PowerPoint presentations or other slides, keywords or diagrams on flip charts and videos to overcome language barriers [11] and to increase trainees’ comprehension of the safety information explained [10]. Previous studies have reported inconsistent results on the effects of training interventions among migrant farmworkers. Indeed, some studies have reported significant positive effects of training in terms of increased farmworkers’ knowledge of OSH issues [12,13], whereas other studies have found no differences between trained and untrained participants [14,15,16,17]. Some authors have interpreted these contrasting results in light of the need to adapt the training materials to an audience of migrants [10,18,19,20], considering particularly the content, layout and style of the graphics [21], to make safety communication more effective. A relevant contribution to reach this aim may come from ergonomics and its user-centred perspective in system design [22]. The ergonomics discipline focuses on users and their capabilities and needs to plan, design and evaluate human–artefact interactions, aiming to promote safety, healthiness and performance in living and working environments. The ergonomic approach is based on users’ active involvement in both the development and evaluation of design solutions that should meet users’ requirements, resulting in usable products, interfaces and services [23,24].

Usability refers to “making sure that something works well” and that any person can use it for its intended purpose without frustration [25] (p. 5). The International Organization for Standardization (ISO) defines usability as “the extent to which a product can be used by specified users to achieve specific goals with effectiveness, efficiency and satisfaction in a specified context of use” [26] (p. 6). Effectiveness measures the goodness of the output by users or whether users can perform their tasks (i.e., completion rate and/or number of errors), whereas efficiency has to do with the quantity of work output in relation to the time, effort and resources involved. User satisfaction is a measure of the user’s attitudes and perceptions about the quality of the interaction with an artefact [27]. Usable artefacts allow users to complete their tasks well, in a timely manner and with minimum frustration. The usability of a system (i.e., a measure of how easy it is to use a system) and its learnability (i.e., how easy it is for users to learn and master the system) have a close relationship, with some authors considering usability as one of the key components of overall system performance [28,29]. The benefits related to the development of user-centred usable artefacts are widely acknowledged in many fields, especially in the industrial and Information and Communication Technology (ICT) sectors [30], whereas to our knowledge, these artefacts have been under-investigated with regard to the development of safety training material, particularly in the agricultural sector. Empirical studies describing a user-centred design of training material for migrant farmworkers are still lacking compared to those concerning other sectors (see, for instance, the study by Evia and Patriarca, [31], focusing on the construction industry). Very few previous studies in which the design of the training materials and tools directly involved migrant farmworkers have shown the increased safety knowledge of these workers after receiving the training [5,7]. However, to assess the efficacy of the intervention, these studies have not typically evaluated the usability of the training material but have only addressed one of its components (the effectiveness) in terms of an increased proportion of correct responses in questionnaires assessing migrant farmworkers’ knowledge of safety issues. The other metrics of the usability of training materials—i.e., efficiency and satisfaction—have been scarcely considered, even though effectiveness, efficiency and satisfaction should be considered independent aspects of usability and all be included in a usability evaluation. The reason for this is that when “researchers or developers use a narrower selection of usability measures for evaluating a system, they either (a) make some implicit or explicit assumptions about the relations between usability measures in the specific context or (b) run the risk of ignoring important aspects of usability” [32] (p. 351). In addition, it should be noted that most of this previous research regarding safety training for migrant farmworkers has dealt with the efficacy of training interventions addressing migrants of Latino/Hispanic origin in a US context [19]. However, the literature has overlooked other cultural contexts, such as Europe, despite it being characterised by a high heterogeneity of cultures, languages and important migration fluxes.

### Context and Aims of the Present Study

Agriculture is one of the most productive sectors with the highest rate of migrant workers in Europe [33], and it is also one of the most hazardous industries [34,35]. Migrant farmworkers are particularly exposed to safety risks, and they report a higher incidence of work-related injuries compared to native workers [20,36]. In Italy, in particular, the migrant agricultural workforce includes approximately 320,000 operators, representing approximately 25% of the non-family agricultural labour force [37]. Previous statistics have shown that between 2013 and 2017, an increasing number of migrants were involved in non-fatal incidents, increasing from 3400–3550, while a decreasing rate was reported for Italian farmworkers, decreasing from 35,000–29,000 [38]. Regarding fatal incidents, in 2015, 193 out of 1246 fatalities involved migrant farmworkers [38]. The presence of migrant farmworkers is particularly relevant in the Piedmont region in northwestern Italy. Indeed, according to the last available statistics, in 2012 in Piedmont, there were 25,312 migrants employed in the agricultural sector [37].

In accordance with the Italian legislative decree 81/08 on the protection of occupational health and safety [39], the employer has to provide appropriate and adequate training to each worker, and it must be easily comprehensible for migrants [39]. Considering these legal requirements and the urgent need to equip migrant farmworkers with clear safety information [40], safety training material that can be comprehensible, with minimum effort and high satisfaction of trainees, could, therefore, provide a valuable contribution to the promotion of the occupational health and safety of the migrant farm workforce.

In the present study, an ergonomic user-centred approach actively involving migrant workers and trainers was adopted to develop safety training material tailored to migrants employed in the Italian agricultural sector. The usability of the new material was then evaluated with a new group of migrant farmworkers by assessing the effectiveness of the material in communicating information (i.e., the pre- and post-training knowledge of safety topics), the efficiency in learning with the new material (i.e., the effort in learning) and participants’ satisfaction with the developed material. The study was conducted in accordance with the Declaration of Helsinki, and the protocol was approved by the Research Advisory Group (RAG) of the Institute for Agricultural and Earthmoving Machines (IMAMOTER) of the National Research Council of Italy (CNR) on 15 November 2016.

## 2. Materials and Methods

### 2.1. Analysis of Users’ Training Needs

At present, based on the regional guidelines developed in accordance with the legislative decree 81/08, in Piedmont, OSH training for farmworkers is held during working hours and is administered by local farmers’ associations or private training agencies [41]. Classes are usually composed of 15–20 migrant farmworkers, and the fundamental requirement for them to participate in the training courses is having passed the mandatory test, which guarantees they have the minimum required proficiency in written and spoken Italian. The safety training material consists of lectures based on an oral presentation supported by slides projected onto a screen. The topics to be addressed are stated by the legislative decree 81/08, and they deal with 12 different topics: an introduction to the important role of safety training and the Italian decree 81/08, who the members of health and safety management are and what their roles are in the workplace (also including employers’ and employees’ duties and rights and fines in cases of non-compliant behaviours both for employers and employees), first aid, behaviour to be adopted and where to turn in case of injury, fire risk, electrical risk, risk of falling, vibration exposure, biological and chemical risks, safety sign comprehension, personal protective equipment (PPE) adoption, handling of heavy loads and farm-machinery-related hazards [39].

To investigate users’ training needs and develop the training material accordingly, a qualitative approach resorting to a series of focus groups was adopted, based on the recommendations by Parker [42], who recognised the focus group as the most effective technique in agriculture to obtain useful feedback from participants, especially in the early stages of a design process [43]. A list of possible migrant participants employed on farms was provided by Italian farmers’ association responsible for health and safety training in the Piedmont region. To be included in the study, migrant farmworkers had to hold stable full-time employment on farms and have attended the mandatory safety training required by the legislative decree 81/08 to be employed in agriculture in Italy during the previous year.

All the participants were informed of the nature of the study and signed an informed consent form. A total of five focus groups, one with six Italian trainers of farmers’ association and four focus groups with 21 migrant farmworkers were performed. The interview guide for the focus group was developed by the research team based on previous studies aimed at identifying perceptions of and feedback on OSH programs addressing migrant farmworkers [44,45,46]. The questions were designed to elicit responses and discussion regarding personal considerations and suggestions about the actual training and how improve it: (1) critical issues about traditional training courses provided by farmers’ association; (2) the most and the least interesting/easy to teach/learn training topics among those stated by the legislative decree 81/08; and (3) suggestions to improve the safety training material (e.g., formats and media, which may help in enhancing the comprehension of different topics).

The focus groups were conducted in Italian by two ergonomists of the research team and were audio-recorded. For each focus group, the recording was transcribed verbatim and then underwent a qualitative content analysis supported by NVivo software v.11 (QSR International, Melbourne, Australia). Two members of the research team categorised participants’ responses. The information, main issues and suggestions raised by the focus groups were used to identify key areas of intervention and users’ requirements for the design of the new training material. Table 1 reports the most significant critical issues reported by participants (both trainers and migrants) about traditional safety training and its topics and suggestions for improving the safety training material. In regard to the critical issues in traditional safety training, both trainers and migrants highlighted some common factors: (1) the difficulties related to the language and the wide use of texts; (2) the scarce effectiveness of oral presentations in conveying safety messages compared to other visual elements (images and videos); and (3) difficulties in remembering specific tasks and contexts in which safety behaviours and safety equipment should be adopted. Of all the topics discussed during the safety training, six topics emerged as the most critical to be taught and/or learned: (1) the members of health and safety management structure and their relationships with workers; (2) the employer’s and employees’ obligations and rights; (3) the use of PPE; (4) farm risks related to fire; (5) electricity-related risks; and (6) machinery-related risks (Table 1).

In regard to the suggestions provided by participants to improve the traditional training material, an increasing number of pictures and images, in terms of both photographs and cartoons, higher involvement during lessons through trainees’ questions and a reduced amount of long texts, emerged as the main themes during the focus groups.

### 2.2. Development of the Safety Training Materials

**Figure 1:**
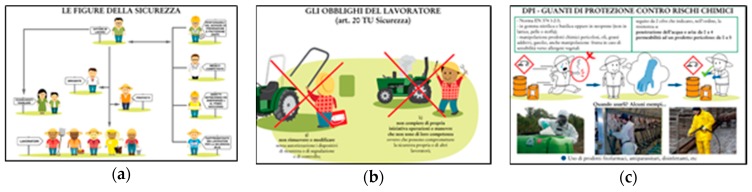
Some of the slides designed for the new training material developed: (**a**) farmworkers’ rights and duties, (**b**)identification and roles of members of health and safety management and (**c**)use of contrast to highlight some details and the primary role as provided by photographs.

Based on the exploration of the critical issues in traditional training material and suggestions for improvement, the training material was developed by taking into consideration specific design criteria and user requirements [47]. The developed graphical training material, which underwent the evaluation, was composed of 75 slides realised in 2D graphics. The visuals depicted those training topics identified as more critical during the focus groups with migrants and trainers in the analysis of training needs. The type of graphical representation used in the new slides was chosen based on the suggestions that emerged during focus groups. Thus, the human figures in the graphical materials presented funny facial expressions and a cartoon style. The features of the characters were defined according to the role that each character plays in the workplace and to facilitate workers’ identification with the depicted human figure. To meet the demand of more images, photographs were used together with pictures and icons to enhance the noticeability of hazards and to facilitate the contextualisation of some hazardous situations. The images represented the main part of the slides, while the text was reduced with the purpose of accompanying the pictures with short sentences and highlighted keywords (e.g., sources of risk in the workplace). The physical/spatial relationship between the text and pictures was designed by taking into account the principle of text parallels [48] to make the text and pictures mutually supportive. Some examples of the developed material are provided in Figure 1.

**Figure 2:**
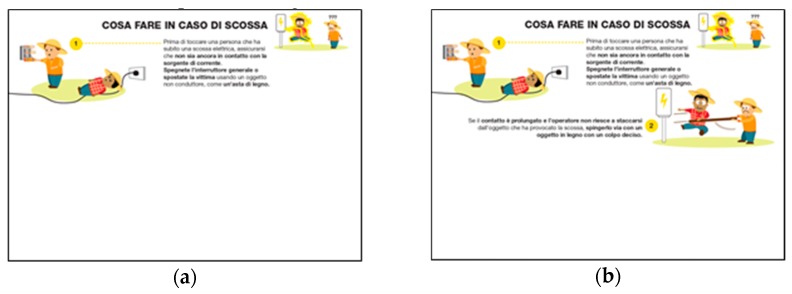

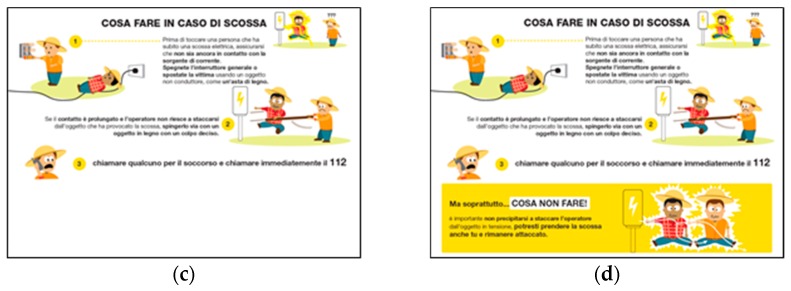
Slides about “what to do in case of electrical shock” shown to migrant farmworkers: a basic animation was added to the slide to explain the correct behaviour to be performed in four different steps: (**a**) turn off the flow of electricity; (**b**) remove the wire or the electrocuting appliance or source with a wooden stick; (**c**) call local emergency; (**d**) do not touch someone who has been shocked if they are still in contact with the source of electricity.

**Figure 3:**
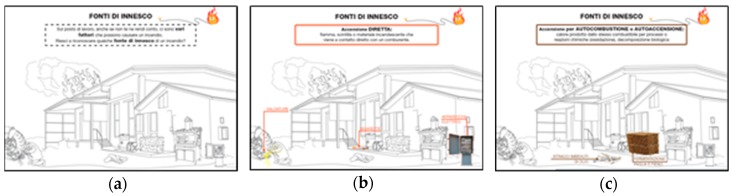
Quiz slide to encourage the involvement of participants in regard to fire risk: (**a**) participants were asked whether they recognised some ignition sources; (**b**) direct ignition source was highlighted; and (**c**) spontaneous ignition source was highlighted.

**Figure 4:**
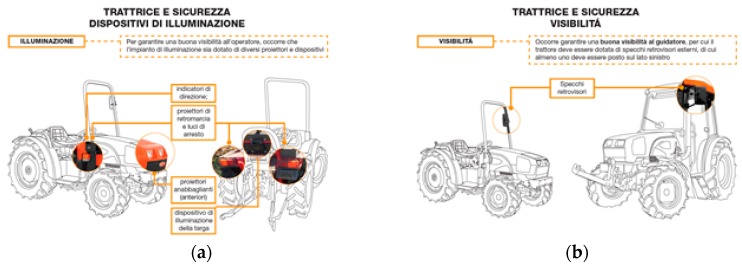
Images developed to focus users’ attention on specific details on the tractor: (**a**) what and where the lighting devices are and (**b**) where the vision devices such as the rear-view mirrors are.

The slides were also characterised by other aspects to increase the interaction between trainers and trainees:

(a) The entire content of the slide was not shown immediately, but basic animations were added to show images step-by-step such as a story described by images (Figure 2);
(b) To make the lecture more engaging, the trainer involved the trainees with some questions during the lesson, and the slides were developed as a visual quiz to encourage the trainees to answer the trainer’s questions (Figure 3 and Figure 4).

### 2.3. Usability Evaluation of the Developed Training Material

The usability of the training material was evaluated in a new group of migrant farmworkers (*n* = 20) by means of an ad hoc paper-and-pencil questionnaire addressing the three metrics defined in the international standard ISO 9241-210 [26]: (1) effectiveness, operationalised as the number of errors in the knowledge of safety topics reported in the slides before and after the training; (2) efficiency, measured in terms of the perceived effort in learning; and (3) satisfaction with the material. No workers from the initial focus groups on users’ requirements were involved in this evaluation. These participants have never received any previous safety and health training in agriculture, but they have already passed the mandatory test required by the Italian OSH regulation, which guarantees a minimum proficiency level in written and spoken Italian to be able to participate in safety training courses.

The questionnaire presented one item for each topic identified as more critical during the focus groups. However, considering participants’ responses in the focus groups, for two topics, a two-fold question was asked. A total of eight multiple-choice items were, therefore, developed, asking participants questions related to the members of health and safety management (who appoint the Prevention and Protection Service Manager (PPSM) and workers’ safety representative, WSR), to workers’ rights and duties (when and if workers can remove equipment protection devices and behaviours that workers must adopt in the case of hazards), to the adoption of PPE (the safety respirator), to the safe behaviour to be adopted in case of fire risk (smoking on the farm), electrical risk (short circuit of electrical equipment) and agricultural-machinery-related risk (removal of the protective guard from the drive shaft). Participants had to choose the correct answer among three possible alternatives. The eight items were administered to all the participants prior to the training to evaluate their naïve knowledge (T0). The training session lasted approximately 2 hours, during which the developed slides were shown to the trainees punctuated by the trainer’s explanations and interaction between the trainer and trainees. Immediately after receiving the training, the same eight items administered at T0 were administered to all participants (T1). For subsequent analysis, the correct answers regarding the knowledge of safety risks were scored as 1, while incorrect responses were scored as 0; a sum score for each participant was then computed, ranging from 0–8. The same scoring procedure was used for the responses given at T0 and T1. Due to the small sample size, knowledge scores at T0 and T1 were then compared through a Wilcoxon signed-rank test. Only at T1 were participants administered two other items referring to perceived effort and satisfaction with the training material. Participants were asked to rate the perceived effort in understanding the safety information presented during the training on a scale ranging from 1 (no effort at all) to 5 (extreme effort). Satisfaction was assessed by asking participants how much they were satisfied with the format in which the safety information was presented during the training session (from 1, not satisfied at all to 5, extremely satisfied). Basic descriptive statistics were then computed for both effort and satisfaction.

## 3. Results of the Usability Evaluation

The main demographic characteristics of the migrant farmworkers involved in the evaluation of the training material are summarised in Table 2.

**Figure 5:**
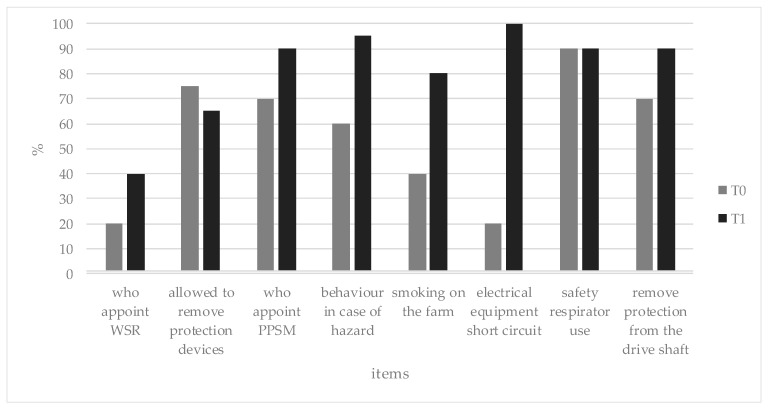
Percentage of correct answers provided by participants at T0 and T1 for each item.

Overall, the response analysis reported an increase in knowledge from T0 to T1. More specifically, the post-test knowledge showed a higher mean percentage of correct answers (81.3%) compared with the mean rate of correct answers reported during the pre-test (55.6%). Concerning the pre-test questionnaire, the items that reported the lowest rate of correct answers were the question related to the appointment of the WSR and that related to the short circuit of electrical equipment (each question reported only 20% correct answers). In contrast, the question related to PPE adoption (safety respirator) yielded a higher percentage of correct answers (90%). Concerning the post-test questionnaire, the lowest score was reported for the appointment of the WSR, even though an increasing rate of correct answers was reported after the training (40%). The question related to electrical risk had the highest comprehension rate (100%). Detailed information regarding the correct answers is reported in Figure 5.

The Wilcoxon test showed that the global knowledge score at T1 was significantly higher than that at T0 (Z = − 3.67, *p* = 0.000). In regard to perceived effort and satisfaction, on the five-point rating scale, the mean effort perceived by participants was 1.50 (SD = 0.89), whereas our participants reported a mean satisfaction of 4.40 (SD = 0.99). Later, at the end of the questionnaire, two participants expressed some opinions regarding the usefulness of the new safety training material by stating the following: “It was very useful; I had some difficulties but I was able to understand thanks to the photos,” and “Everything seemed useful; there was not a single interesting thing, and everything was useful for understanding how I can protect myself at work”.

## 4. Discussion

The diversity of the language, culture and education of migrant workers requires dynamic and creative approaches to the design of occupational safety training materials [10,49]. In this study, we adopted a user-centred ergonomic approach in which the target users were actively involved during both the development and the evaluation of new training material. Consistent with Parker [42], the adoption of the focus group was shown to be an effective method for involving users and allowing for a wealth of information to be collected [43] to highlight criticalities and suggestions for the design of the new training material.

The ergonomic approach adopted in the present study led to a training tool based mainly on visual communication, which appeared to be more usable compared to the traditional, mainly written, training tool [50]. The better user experience associated with the new training material supports the advantages of the active involvement of users and the consideration of their variability in needs and expectations both in the design and evaluation of a product [43]. As noted by Arning et al. [51], the comprehensibility of graphical systems is essential for providing noticeable and effective communication and transferring the relevant knowledge into the work process. The present result is consistent with the evidence of Neitzel et. al. [52], who reported that traditional written safety and health training materials may not be universally understood, whereas images and videos can be a more effective form of communication. Following participants’ suggestion to redesign the training material by adopting more images and different types of graphic visual elements, in the material developed for the present study, the written text was used as support for the images, enriching them with additional and contextualised information [51]. This issue may be further investigated by arranging an experimental study in which participants have to rate the comprehensibility of the safety information provided through different combinations of text and images, similar to what has been done by Wilkinson et al. [53] in their study on pesticide safety information.

The responses provided by participants during the different focus groups in the early stages of the project are consistent with the literature in which visual elements, both photographs [54] and non-photorealistic images [55], are preferable and more comprehensible than written text. Although some participants reported appreciating the adoption of videos during the safety training lectures, some other responses by both trainers and trainees mirrored previous evidence in which videos and animations were difficult to manage (i.e., are fleeting and avoiding stopping it frequently) [56]. After the training, participants significantly improved their knowledge about the topics identified as more critical during the initial analysis of user needs. This result is consistent with the increased safety knowledge observed in previous studies in which migrants were directly involved in the design of training interventions [5], and it is promising if we consider that the knowledge of health risks and benefit of safety practices create the precondition to carry out a safe behaviour [57,58].

Participants trained with the new graphical material reported low levels of perceived effort (mean of 1.5 out of 5) and high levels of satisfaction (mean of 4.4 out of 5) in understanding the topics presented during the course. This is a relevant result since the mental effort required to accomplish a task represents a critical variable in the overall system performance: the higher users’ mental effort is, the greater the probability that an error occurs [59]. Attention, fatigue and working memory are known to influence the noticeability, encoding and comprehension of warning messages [60,61]; therefore, these variables warrant a more detailed investigation in the field of safety training to point out the most efficient materials and methods to use to increase participants’ comprehension of safety issues. In addition, the increase in safety knowledge that emerged in the present study is consistent with that reported by Burke et al. [1], in which information on screen and supporting safety material could reinforce less engaging methods such as lectures and could obtain positive results in terms of user knowledge.

Some limitations of the present study should be taken into account. First, the research involved a group of migrants employed in a specific area of one Italian region. Addressing participants from a local area allowed us to have similar background contexts (at least regarding migrants’ work experience in Italy) and more comparable data [62]; however, in the future, it would be interesting to extend the study to additional European areas with a high rate of the migrant agricultural workforce. Furthermore, as pointed out by Brunette [63], training interventions developed for one ethnic group may not have the same effect among workers from other countries. Due to the small sample size, in this study, we could not consider the possible effects of nationality: further development of the ergonomic approach adopted in the present study may include the involvement of migrant farmworkers recruited by area of origin (i.e., differentiating by continent and cultural/linguistic area) to design different training materials, one for each ethnic group, to better fit the cultural peculiarities of each group.

In a further development of the research, some objective measures of the efficiency of the training material may be collected, for instance, by recording the time spent by participants in answering the targeted questions. Finally, even though safety knowledge is an important precondition for safe behaviour [57], some previous research [64] has shown that an increase in safety knowledge is not always followed by an increase in safe behaviours in the workplace. Therefore, in future research, it would be useful to investigate not only the effectiveness of the training material in conveying the targeted information (i.e., enhancing safety knowledge) but also the effectiveness of the training itself, assessing on-the-job transfers [65], in terms of migrant farmworkers’ behavioural compliance with safety practices, after being trained with the material developed by means of the ergonomic approach. A longitudinal assessment of training effectiveness could be performed by collecting six-month follow-up data on the actual injury rates of trained workers (as was done in [66]), which would give rise to significant considerations about training effectiveness and suggestions for further interventions.

## 5. Conclusions

The promotion of occupational safety and health through training is a difficult process for migrant workers because of language and cultural differences, which may prevent the correct comprehension of safety information. In this study, we addressed the population of migrant farmworkers, who appear to be overlooked by the literature related to OSH training in the European context, despite the high rate of foreign workforce in European countries, especially in the agricultural sector. The results of the present study showed that developing training material by means of the involvement of target users can lead to the more effective and efficient communication of safety information among migrants of different origins compared to traditional material. This evidence is in line with the requirements of both the Italian OSH legislation and the international convention on OSH in agriculture, which state that the employer has to provide appropriate and adequate training to each worker, and such training must be easily comprehensible for migrants [39]. In addition to providing a valuable method for evaluating the overall performance of the new training material, the ergonomic approach used in the present study allowed users to also engage during the development phase of the training material, leading to a training tool that was not only more engaging but also tailored to the specific context of use and to the requirements of the specific population of users.

Although the approach developed and presented here could be improved and the usability of the developed material should be further investigated with wider samples of migrants, the present analysis suggests the critical need to disseminate this and other similar findings to the research community and other organisations that promote occupational safety and health for all, not only in agriculture but also in other high-risk industries, to point out the importance of actively involving final users when developing safety training interventions.

## Figures and Tables

**Table 1:** Main comments of trainers and migrant farmworkers.

Topics in the Interview Guide	Elicited Topics in the Training	Group of Respondents	Key Comments for Training Material Implementation
Criticalities of the traditional safety training	Overall comments	Migrants	Too much information provided by trainers during lectures does not allow most learners to retain the content of the training for more than one week.
	The decree 81/08 (related to members of the health and safety management structure and risks from agricultural machinery)	Trainers	The less engaging parts of the training material are those reporting legislative definitions, concepts of risk perception and how the different parts of agricultural machinery work.
		Migrants	Migrants just remember the number of the decree and the overall subject, but related content results are difficult to remember due to the enormous number of slides and the numerous definitions.
	Language and style of communication	Trainers	The adoption of too many technical and difficult words should be avoided, and simpler speech should be preferred.
		Migrants	In the training material, there are too many written parts with too many unfamiliar words.
Topics in traditional training: the least/most comprehended topics and interesting topics	The decree 81/08 and who the safety and health management figures are	Migrants	Migrants just recall the role of two figures: the PPSM^1^ and the WSR^2^.
	PPE^3^ adoption and working safely with agricultural equipment and machinery	Migrants	Migrants find the topic of PPE^3^ adoption truly interesting, and a number of them remember the importance of wearing suitable PPE^3^ (gloves, suit, safety glasses and safety shoes) during working hours.
	Fire risk	Migrants	The part of the training material related to fire risk contains words and definitions that are too difficult to understand.
	Electrical risk	Trainers	The part of the training material related to electrical risk should be redesigned to reduce the number of slides and make the content more comprehensible.
Suggestions for improving the safety training presentation (e.g., preferred formats and media)	Overall comments	Trainers	Lectures need to be streamlined to keep learners’ attention level high. guifenSafety figures should be represented in a different way, avoiding the bullet points currently used, for instance, adopting a cartoon style.
		Migrants	Migrants report remembering more information through the use of images rather than speech, and thus, the use of more images (photographs or cartoons) is encouraged.guifenTrainers should be more involved during lectures, for instance, addressing direct questions to learners.

^1^ PPSM (Prevention and Protective Service Manager); ^2^ WSR (Workers’ Safety Representative); ^3^ PPE (Personal Protection Equipment).

**Table 2:** Demographic characteristics of the migrant farmworkers trained with the new material.

		Intervention Group	
Variable	Level	*n*	%
Gender	Male	20	100
		**Mean (SD)**	
Age (years)		36.50 (10.46)	
Education (years)		9.35 (2.32)	
Length of stay in Italy (years)		8.33 (4.80)	
